# Rational Design of Antibiotic Treatment Plans: A Treatment Strategy for Managing Evolution and Reversing Resistance

**DOI:** 10.1371/journal.pone.0122283

**Published:** 2015-05-06

**Authors:** Portia M. Mira, Kristina Crona, Devin Greene, Juan C. Meza, Bernd Sturmfels, Miriam Barlow

**Affiliations:** 1 School of Natural Science, University of California Merced, Merced, California, United States of America; 2 Department of Mathematics and Statistics, American University, Washington, DC, United States of America; 3 Departments of Mathematics, Statistics, and EECS, University of California, Berkeley, California, United States of America; Columbia University, UNITED STATES

## Abstract

The development of reliable methods for restoring susceptibility after antibiotic resistance arises has proven elusive. A greater understanding of the relationship between antibiotic administration and the evolution of resistance is key to overcoming this challenge. Here we present a data-driven mathematical approach for developing antibiotic treatment plans that can reverse the evolution of antibiotic resistance determinants. We have generated adaptive landscapes for 16 genotypes of the TEM β-lactamase that vary from the wild type genotype “TEM-1” through all combinations of four amino acid substitutions. We determined the growth rate of each genotype when treated with each of 15 β-lactam antibiotics. By using growth rates as a measure of fitness, we computed the probability of each amino acid substitution in each β-lactam treatment using two different models named the Correlated Probability Model (CPM) and the Equal Probability Model (EPM). We then performed an exhaustive search through the 15 treatments for substitution paths leading from each of the 16 genotypes back to the wild type TEM-1. We identified optimized treatment paths that returned the highest probabilities of selecting for reversions of amino acid substitutions and returning TEM to the wild type state. For the CPM model, the optimized probabilities ranged between 0.6 and 1.0. For the EPM model, the optimized probabilities ranged between 0.38 and 1.0. For cyclical CPM treatment plans in which the starting and ending genotype was the wild type, the probabilities were between 0.62 and 0.7. Overall this study shows that there is promise for reversing the evolution of resistance through antibiotic treatment plans.

## Introduction

Antibiotic resistance is an inevitable outcome whenever antibiotics are used. There are many reasons for this: 1) As humans (also as eukaryotes), we are vastly outnumbered by bacteria in nearly all measures, including total population size, biomass, genetic diversity, emigration, and immigration [1]; 2) bacteria can use horizontal gene transfer to share resistance genes across distantly related species of bacteria, including non-pathogens [2]; 3) compared to humans, bacteria have relatively few vulnerable target sites [3]; 4) microbes are the sources of nearly all antibiotics that are used by humans [4]. Given the overwhelming numbers of bacteria, the limited number of target sites, the numerous ways that they can infect humans, and that they have been exposed to naturally occurring antibiotics for billions of years, resistance to antibiotics used by human populations is unavoidable.

Once resistance is present in a bacterial population, it is exceedingly difficult to remove for several reasons. If any amount of antibiotic is present in the environment, antibiotic resistance genes will confer a large fitness advantage [5], and even when antibiotics are not present in an environment, the fitness costs for carrying and expressing resistance genes are small to non-existent [6]. In addition to it being difficult to remove antibiotics from the environment [7], even if humans were to completely abandon the use of antibiotics, resistance would persist for years [8].

Efforts to remove resistance genes from clinical environments by either discontinuing or reducing the use of specific antibiotics for some period of time, either through general reduction of antibiotic consumption or periodic rotations of antibiotics (cycling) have not worked in any reliable or reproducible manner [9]; indeed it would have been surprising if they had worked [10],[11].

Since antibiotic resistance *is* unavoidable, it only makes sense to accept its inevitability and develop methods for mitigating the consequences. One reasonable approach is to rotate the use of antibiotics. This has been implemented in many ways and there are recent studies to model the optimal duration, mixing versus cycling, and how relaxed antibiotic cycles may be and still function as planned [12, 13]. However, none of those models have focused on developing a method for designing an optimal succession of antibiotics.

In a previous publication [14], we proposed that susceptibility to antibiotics could be restored by rotating consumption of multiple antibiotics that are a) structurally similar, b) inhibit/kill bacteria through the same target site, and c) result in pleiotropic fitness costs that reduce the overall resistance of bacteria to each other. We presented a proof-of-principle example [14] of how this might work with a series of β-lactam antibiotics in which some of them would select for new amino acid substitutions in the TEM β-lactamase and others that would select reversions in TEM ultimately leading back to the wild type (un-mutated) state. We have focused particularly on β-lactamases because there is often no fitness cost associated with their expression, and they are particularly difficult to remove from clinical microbial populations.

Our current work seeks to identify β-lactam treatment plans that have the highest probability of returning a population expressing a small number of variant TEM genotypes to the wild type state. The wild type TEM-1, and a handful of its descendants, confers resistance to penicillins alone. However, most of the descendants confer resistance to either cephalosporins or penicillins combined with β-lactamase inhibitors (inhibitor resistance), and a few confer resistance to both. Of the 194 clinically identified TEM genotypes that encode unique amino acid sequences [15], 174 (89.7%) differ from the wild type TEM-1 by at most four amino acid substitutions (see Table 1). Our choice of a system that includes four amino acid substitutions is based upon an apparent threshold for amino acid substitutions among functional TEM genotypes. The rarity of the co-existence of cephalosporin resistance and inhibitor resistance and the fact that no single substitution confers both phenotypes suggested that sign epistasis (i.e. reversals of substitutions from beneficial to detrimental) exists as the substitutions that contribute to this dual phenotype are combined. We have assumed that substitutions arise according to the strong selection weak mutation model (SSWM) [16] in which single substitutions reach fixation before the next substitution occurs. Recent work [17] in addition to past phylogenetic analysis [18] and competition experiments [19] suggest that this is a valid model for TEM evolution.

**Table 1:** Distribution of substitutions among TEM enzymes.

Number of amino acid substitutions	Number of identified TEM genotypes
1	53
2	53
3	37
4	31
5	10
6	2
7	2
8	0
9	0
10	1
11	1

The ability to apply selective pressures that favor reversions of substitutions within an evolved TEM genotype would increase the number of antibiotics that could be used. To embark upon our effort of determining the best way to do this, we decided to create a model system based upon the TEM-50 genotype, which differs from TEM-1 by four amino acid substitutions. All four substitutions by themselves confer clearly defined resistance advantages in the presence of certain antibiotics. Additionally, TEM-50 is one of the few genotypes that simultaneously confers resistance to cephalosporins and inhibitor combined therapies.

## Results

### From experimental data to mathematical models

We created all 16 variant genotypes of the four amino acid substitutions found in TEM-50 using site directed mutagenesis (Table 2) and measured the growth rates of 12 replicates of *E*.*coli* DH5α-E expressing each genotype in the presence of one of fifteen β-lactam antibiotics (Table 3). Each genotype was grown in each antibiotic in 12 replicates. We computed the mean growth rate of those replicates (Table 4) and the variance of each sample, as well as the significance between adjacent genotypes that differed by one amino acid substitution. This was done using one-way ANOVA analysis.

**Table 2:** Variant Genotypes Created, Binary Codes, Substitutions and (Names of Genotypes Identified in Clinical Isolates).

Number of Substitutions	Binary Genotype Code	Genotypes with substitutions found in TEM-50
0	0000	No substitutions, (TEM-1)
1	1000	M69L, (TEM-33)
1	0100	E104K, (TEM-17)
1	0010	G238S, (TEM-19)
1	0001	N276D, (TEM-84)
2	1100	M69L, E104K, (Not identified)
2	1010	M69L, G238S, (Not identified)
2	1001	M69L, N276D, (TEM-35)
2	0110	E104K, G238S, (TEM-15)
2	0101	E104K, N276D, (Not identified)
2	0011	G238S, N276D, (Not identified)
3	1110	M69L, E104K, G238S, (Not identified)
3	1101	M69L, E104K, N276D, (Not Identified)
3	1011	M69L, G238S, N276D, (Not identified)
3	0111	E104K, G238S, N276D, (Not identified)
4	1111	M69L, E104K, G238S, N276D, (TEM-50)

**Table 3:** β-lactam Antibiotics used for this study.

β-lactam Antibiotic	FDA approval	Antibiotic Group
Ampicillin (AMP)	1963	Aminopenicillin
Amoxicillin (AM)	1972	Aminopenicillin
Cefaclor (CEC)	1979	Cephalosporin
Cefotaxime (CTX)	1981	Cephalosporin
Ceftizoxime (ZOX)	1983	Cephalosporin
Cefuroxime (CXM)	1983	Cephalosporin
Ceftriaxone (CRO)	1984	Cephalosporin
Amoxicillin + Clavulanic acid (AMC)	1984	Penicillin derivative + β-Lactamase inhibitor
Ceftazidime (CAZ)	1985	Cephalosporin
Cefotetan (CTT)	1985	Cephalosporin
Ampicillin + Sulbactam (SAM)	1986	Penicillin derivative + β-Lactamase inhibitor
Cefprozil (CPR)	1991	Cephalosporin
Cefpodoxime (CPD)	1992	Cephalosporin
Pipercillin + Tazobactam (TZP)	1993	Penicillin derivative + β-Lactamase inhibitor
Cefepime (FEP)	1996	Cephalosporin

**Table 4:** Average Growth Rates (x 10^–3^): the rows are the fitness landscapes.

	0000	1000	0100	0010	0001	1100	1010	1001
**AMP**	1.851	1.570	2.024	1.948	2.082	2.186	0.051	2.165
**AM**	1.778	1.720	1.448	2.042	1.782	1.557	1.799	2.008
**CEC**	2.258	0.234	2.396	2.151	1.996	2.150	2.242	0.172
**CTX**	0.160	0.185	1.653	1.936	0.085	0.225	1.969	0.140
**ZOX**	0.993	1.106	1.698	2.069	0.805	1.116	1.894	1.171
**CXM**	1.748	0.423	2.940	2.070	1.700	2.024	1.911	1.578
**CRO**	1.092	0.830	2.880	2.554	0.287	1.407	3.173	0.540
**AMC**	1.435	1.417	1.672	1.061	1.573	1.377	1.538	1.351
**CAZ**	2.134	0.288	2.042	2.618	2.656	2.630	1.604	0.576
**CTT**	2.125	3.238	3.291	2.804	1.922	0.546	2.883	2.966
**SAM**	1.879	2.198	2.456	0.133	2.533	2.504	2.308	2.570
**CPR**	1.743	1.553	2.018	1.763	1.662	0.223	0.165	0.256
**CPD**	0.595	0.432	1.761	2.604	0.245	0.638	2.651	0.388
**TZP**	2.679	2.709	3.038	2.427	2.906	2.453	0.172	2.500
**FEP**	2.590	2.067	2.440	2.393	2.572	2.735	2.957	2.446
	**0110**	**0101**	**0011**	**1110**	**1101**	**1011**	**0111**	**1111**
**AMP**	2.033	2.198	2.434	0.088	2.322	0.083	0.034	2.821
**AM**	1.184	1.544	1.752	1.768	2.247	2.005	0.063	2.047
**CEC**	2.230	1.846	2.648	2.640	0.095	0.093	0.214	0.516
**CTX**	2.295	0.138	2.348	0.119	0.092	0.203	2.269	2.412
**ZOX**	2.138	2.010	2.683	1.103	1.105	0.681	2.688	2.591
**CXM**	2.918	2.173	1.938	1.591	1.678	2.754	3.272	2.923
**CRO**	2.732	0.656	3.042	2.740	0.751	1.153	0.436	3.227
**AMC**	0.073	1.625	1.457	1.307	1.914	1.590	0.068	1.728
**CAZ**	2.924	2.756	2.688	2.893	2.677	1.378	0.251	2.563
**CTT**	3.082	2.888	0.588	3.193	3.181	0.890	3.508	2.543
**SAM**	0.083	2.437	0.094	2.528	3.002	2.886	0.094	3.453
**CPR**	2.042	2.050	1.785	1.811	0.239	0.221	0.218	0.288
**CPD**	2.910	1.471	3.043	0.963	0.986	1.103	3.096	3.268
**TZP**	2.528	3.309	0.141	0.609	2.739	0.093	0.143	0.171
**FEP**	2.652	2.808	2.832	2.796	2.863	2.633	0.611	3.203

**Fig 1:**
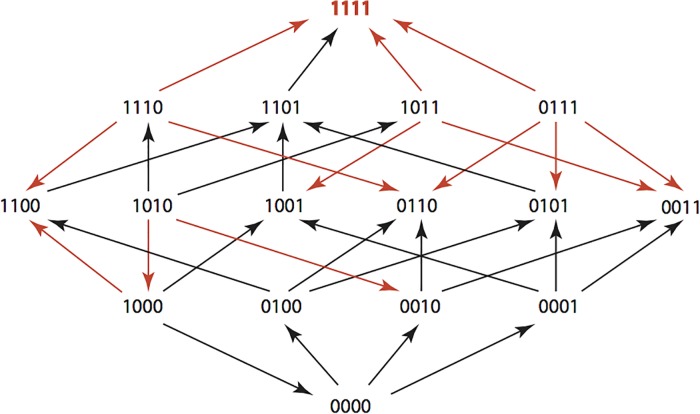
AMP: Ampicillin 256 μg/ml.

**Fig 2:**
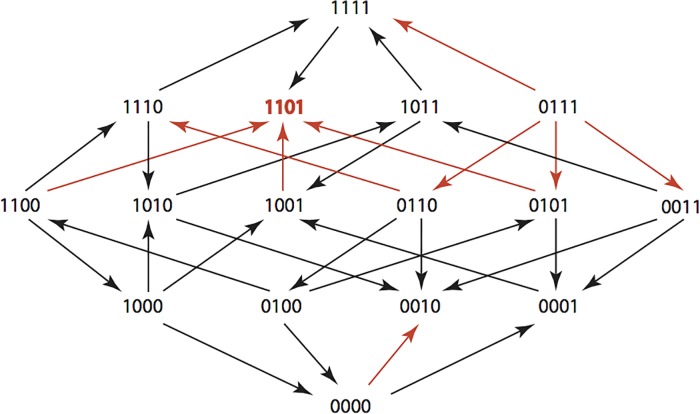
AM: Amoxicillin 512 μg/ml.

**Fig 3:**
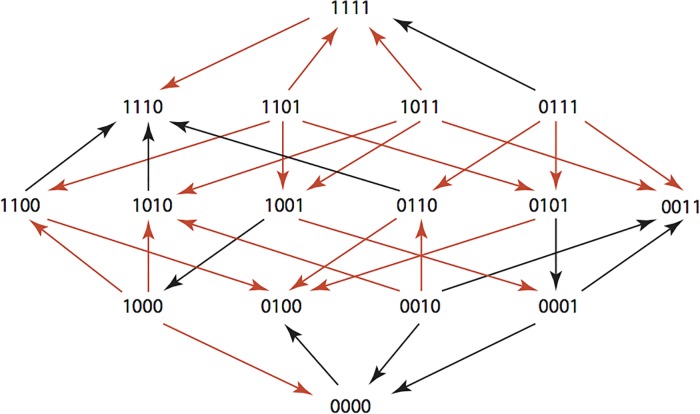
CEC: Cefaclor 1 μg/ml.

**Fig 4:**
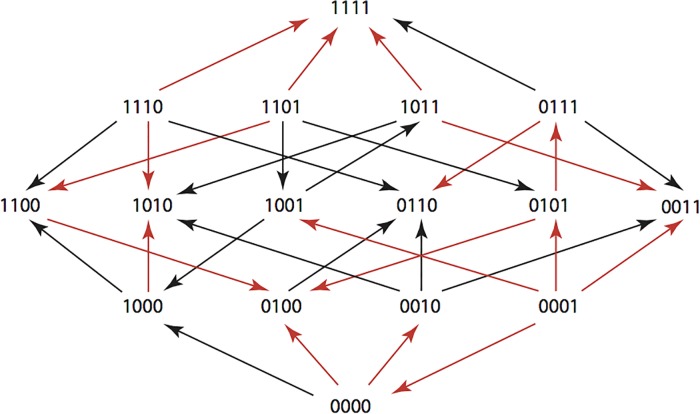
CTX: Cefotaxime 0.05 μg/ml.

**Fig 5:**
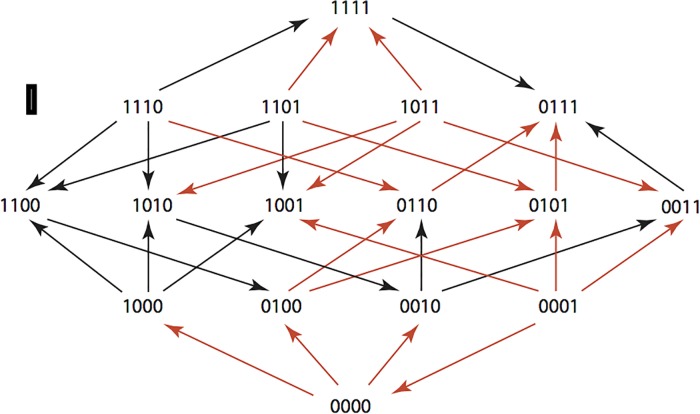
ZOX: Ceftizoxime 0.03 μg/ml.

**Fig 6:**
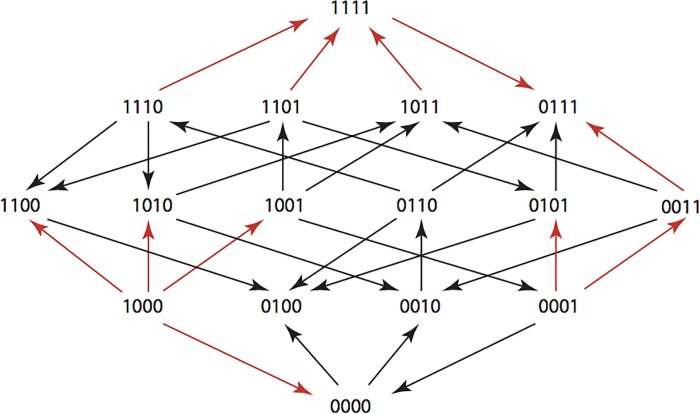
CXM: Cefuroxime 1.5 μg/ml.

**Fig 7:**
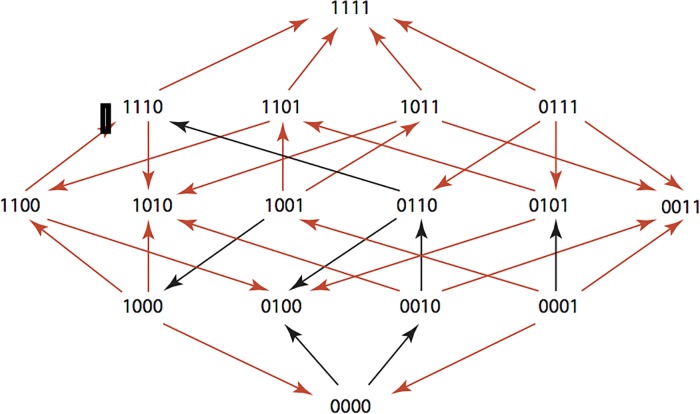
CRO: Ceftriaxone 0.045 μg/ml.

**Fig 8:**
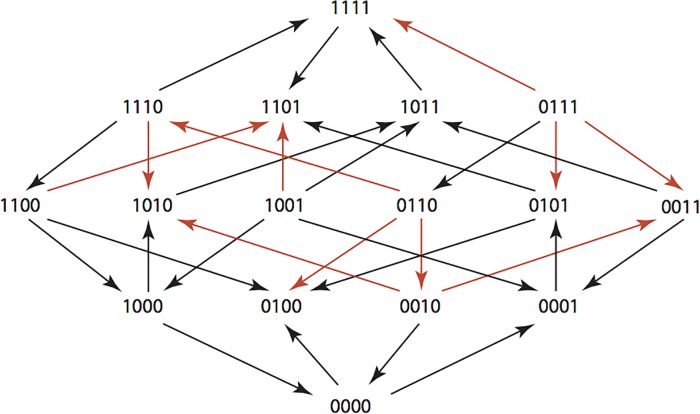
AMC: Amoxicillin/Clavulanate 512 μg/ml and 8μg/ml.

**Fig 9:**
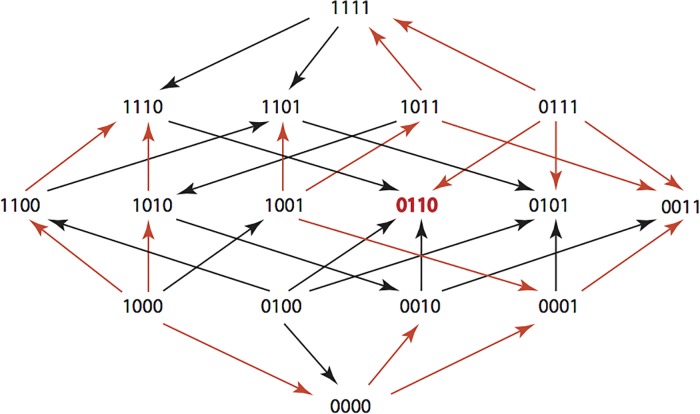
CAZ: Cefazidime 0.1 μg/ml.

**Fig 10:**
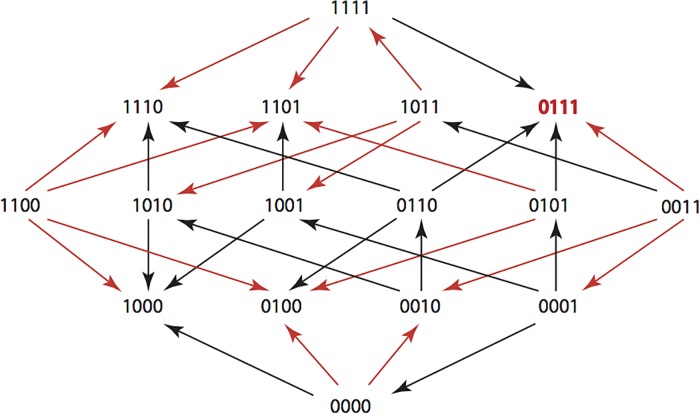
CTT: Cefotetan 0.312 μg/ml.

**Fig 11:**
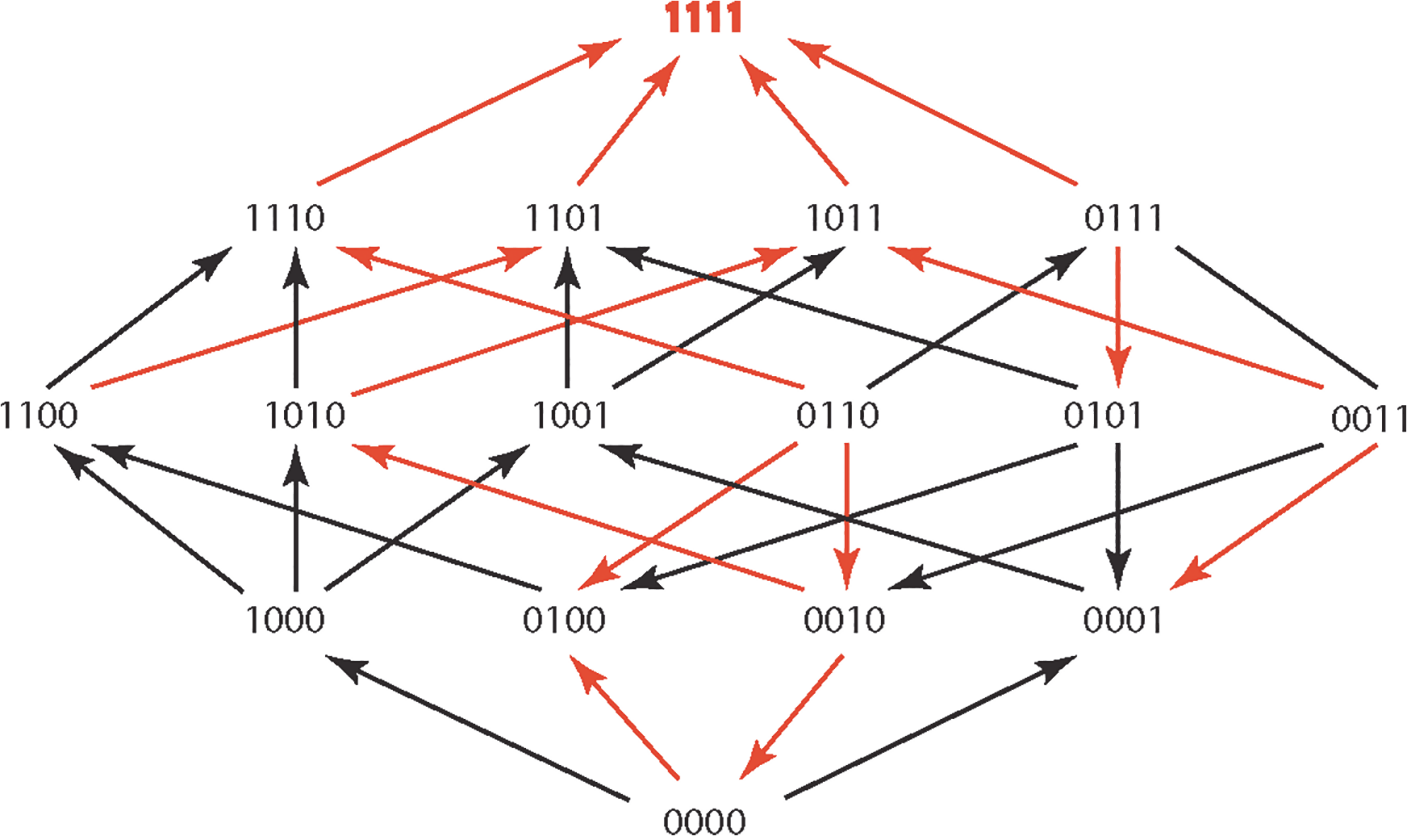
SAM: Ampicillin/Sulbactam 8 μg/ml and 8μg/ml.

**Fig 12:**
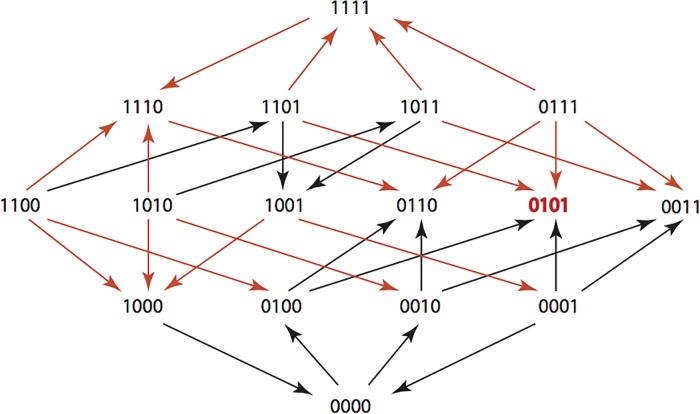
CPR: Cefprozil 100 μg/ml.

**Fig 13:**
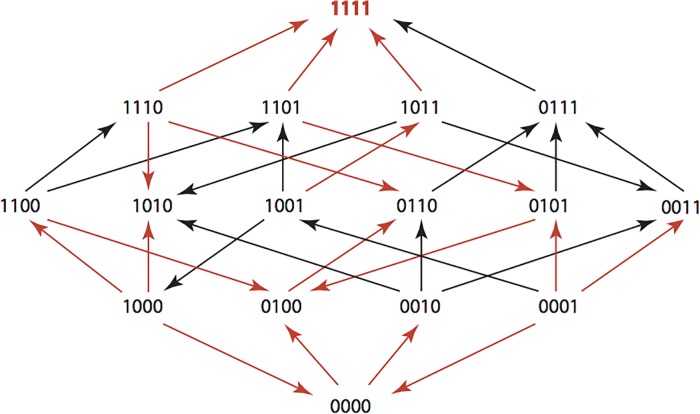
CPD: Cefpodoxime 2 μg/ml.

**Fig 14:**
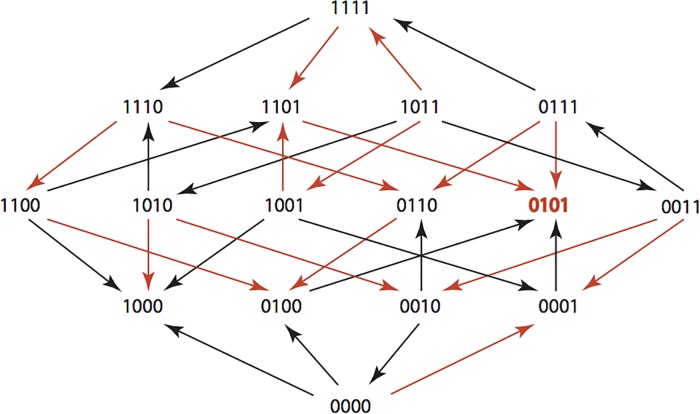
TZP: Pipercillin / Tazobactam 8.12μg/ml and 8 μg.ml.

**Fig 15:**
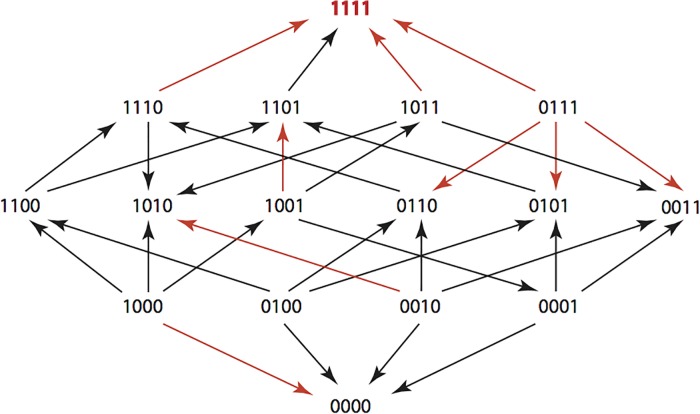
FEP: Cefepime 0.0156μg/ml.

The results are summarized in Figs 1–15, where the arrows in the fitness graphs connect pairs of adjacent genotypes. For each comparison of adjacent genotypes, we indicate the one whose expression resulted in the faster growth by directing the arrowhead towards that genotype, and implying that evolution would proceed in that direction if the two genotypes occurred simultaneously in a population [20, 21]. In other words, the node indicated by the arrowhead would increase in frequency and reach fixation in the population, while the other would be lost. Red arrows indicate significance, and black arrows indicate differences that were not statistically significant by ANOVA, but that may still exist if a more sensitive assay was used.

**Figs 1–15.** These figures present fitness graphs, which are a visual summary of the adaptive landscape 2x2x2x2 tensors in which each resistance phenotype conferred by each TEM genotype is enumerated. Arrows pointing upward represent selection for the addition of a substitution. Arrows pointing downward represent selection for reversions. Red arrows indicate significance between adjacent growth rates as determined by one way ANOVA. Genotypes that confer the most resistance to each antibiotic are shown in red.

We rank ordered the genotypes (Table 5) in each landscape diagram with a score from 1 to 16, with the genotype promoting the fastest growth receiving a score of “1” and the genotype with the slowest growth a score of “16”. This analysis shows that all genotypes have a score of 5 or better and a score of 13 or worse, in at least one landscape, indicating that there is abundant pleiotropy as antibiotic selective pressures change. That pleiotropy provides a basis for effectively alternating antibiotic to restore the wild type.

**Table 5:** Rank Order of Genotypes in Each β-Lactam Antibiotic (Derived From Table 4).

Antibiotic	0000	1000	0100	0010	0001	1100	1010	1001	0110	0101	0011	1110	1101	1011	0111	1111
AMP	11	12	9	10	7	5	15	6	8	4	2	13	3	14	16	1
AM	8	11	14	3	7	12	6	4	15	13	10	9	1	5	16	2
CEC	4	12	3	7	9	8	5	14	6	10	1	2	15	16	13	11
CTX	11	10	7	6	16	8	5	12	3	13	2	14	15	9	4	1
ZOX	14	11	8	5	15	10	7	9	4	6	2	3	12	16	1	3
CXM	11	16	2	7	12	8	10	15	4	6	9	14	13	5	1	3
CRO	10	11	4	7	16	8	2	14	6	13	3	5	12	9	15	1
AMC	9	10	3	14	6	11	7	12	15	4	8	13	1	5	16	2
CAZ	10	15	11	8	6	7	12	14	1	3	4	2	5	13	16	9
CTT	12	3	2	10	13	16	9	7	6	8	15	4	5	14	1	11
SAM	12	11	8	13	5	7	10	4	16	9	14	6	2	3	15	1
CPR	7	9	3	6	8	13	16	11	2	1	5	4	12	14	15	10
CPD	13	14	7	6	16	12	5	15	4	8	3	11	10	9	2	1
TZP	6	5	2	10	3	9	12	8	7	1	15	11	4	16	14	13
FEP	10	15	13	14	11	7	2	12	8	5	4	6	3	9	16	1
Best value	4	3	2	3	3	5	2	4	1	1	1	2	1	3	1	1
Worst value	14	16	14	14	16	16	15	15	16	13	15	14	15	16	16	13
Median value	10	11	7	7	9	8	7	12	6	6	4	6	5	9	15	2

Based on the strong patterns of pleiotropy we observed, we reasoned that the choice and the succession of antibiotics were at least as important as other cycling considerations. We formalized our approach to identifying optimal antibiotic treatment paths as follows.

We considered the 15 antibiotics previously mentioned in Table 3: AMP, AM, CEC, CTX, ZOX, CXM, CRO, AMC, CAZ, CTT, SAM, CPR, CPD, TZP, and FEP and their interactions with a bi-allelic 4-locus TEM system {0,1}^4^ where four functionally important amino acid residues involved in the evolution of TEM-50 are considered. The number "1" denotes an amino acid substitution, whereas "0" denotes no substitution at the site. We experimentally determined growth rates for all genotypes in our TEM system at a selected concentration of each antibiotic. Those growth rates depend upon the states of the four amino acid residues. The growth rates for all genotypes in one antibiotic can be represented by a real 2×2×2×2 tensor *f* = (*f*_*ijkl*_), where *f*(*a*_*r*_) is the fitness landscape for the antibiotic *r*. We can identify *f*(*a*_*r*_) with a vector whose coordinates are indexed by {0,1}^4^. The resulting 15 vectors, one for each antibiotic, are the rows in Table 4.

Our substitution model *M*(*f*) is a function $M:{\mathbb{R}}^{16}\to {\mathbb{R}}^{16\times 16}$ that assigns a transition matrix to each fitness landscape. The rows and columns of *M*(*f*) are labeled by the genotypes {0,1}^4^ according to the degree lexicographic order. The entries in *M*(*f*(*a*_*r*_))_*u*,*v*_ represent the probability that that genotype *u* is replaced by genotype *v* under the presence of antibiotic *a*_*r*_. For that reason, the rows of our transition matrices have nonnegative entries and their rows sum to 1.

We require that our transition matrices respect the adjacency structure of the 4-cube, that is, *M*(*f*)_*u*,*v*_ = 0 unless *u* and *v* are vectors in {0,1}^4^ that differ in at most one coordinate. In other words, we reasoned that resistant strains are most likely to be in competition with those that express resistance genotypes that are immediately adjacent (vary by a single amino acid substitution).

The combined effect of a sequence *a*_1_,…,*a*_*k*_ of *k* antibiotics is described by a new transition matrix

$$M\left(f_{a_{1}}\right)\cdot M\left(f_{a_{2}}\right)\cdot ...\cdot M\left(f_{a_{k}}\right)$$

obtained as the product of the transition matrices for each drug.

For any genotype u other than 0000, our goal is to find a sequence of antibiotics which maximizes the probability of returning to the wild type. In other words, if we restrict to sequences of length k our goal is to find a sequence of antibiotics *a*_1_,…,*a*_*k*_ which maximizes the matrix entry *M*(*f*(*a*_1_))·*M*(*f*(*a*_2_))·…·*M*(*f*(*a*_*k*_))_*u*,0000_. For each *u* this requires searching over all 15^*k*^ antibiotic sequences of length *k*.

### Finding optimal sequences of antibiotics

We used two substitution models to determine the optimal (highest probability) sequences of β-lactams for returning TEM genotypes back to their wild type state. Briefly, the Correlated Probability Model (CPM) allows probabilities to be based upon the actual growth rates. It is given by applying Eq (3) to the growth rates in Table 4. The Equal Probability Model (EPM) assumes that beneficial substitutions are equally likely and that only the direction of the arrows in Figs 1–15 is important. This means that the matrix entry *M*(*f*)_*u*,*v*_ is 1/*N* if genotype *u* has *N* outgoing arrows and there is an arrow from *u* to *v*.

**Fig 16:**
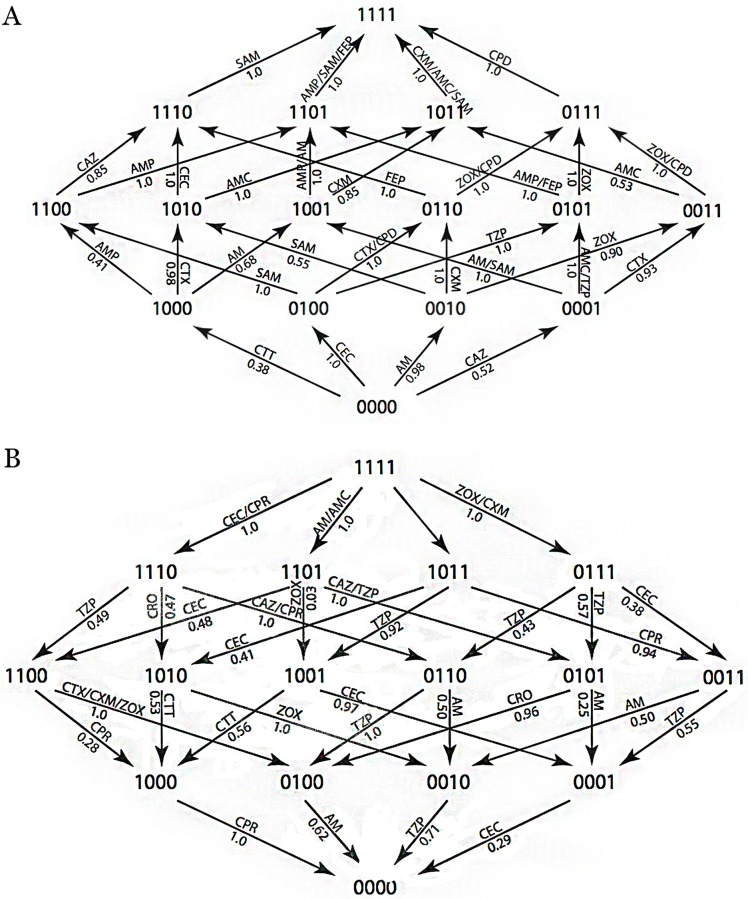
Summary of CPM Substitutions with the Highest Probabilities. Each arrow is labeled by the drug or drugs corresponding to the maximal transition probability, taken over all 15 drugs. Each arrow is also labeled by the maximal probability. The top panel shows which antibiotics selected the addition of substitutions and the bottom panel shows which antibiotics selected reversions. Unlabled arrows are those with low probabilities across all antibiotics.

A visual summary of the highest probabilities according to the CPM is provided in Fig 16. The CPM provides good estimates if fitness differences between genotypes are small [14, 22–24]. The EPM has been used in settings where only rank order (as in Table 5) is available [25].

From the graph, it is possible to find candidate treatment plans. For example, when starting at genotype 1010 the graph shows that the probability for ending at 0000 is 0.71for the sequence ZOX-TZP (0.71 is the product of the arrow labels). Similarly, when starting at 1111 the probability for ending at 0000 is 0.62 for the sequence CEC-CAZ-TZP-AM. When starting at 0001 the graphs shows that a single drug gives probability at most 0.29, whereas the probability for ending at 0000 for the sequence AMC-CRO-AM (one arrow up, two arrows down) is at least 0.96·0.62 = 0.6.

This graph can also be used to generate treatment paths that start and end at the same genotype, making possible the development of a fixed treatment plan. For example, from a starting point 0000, the probability for ending at 0000 is 0.62 for the sequence: CEC-CTX-ZOX-CPD-CPR-CAZ-TZP-AM

**Fig 17:**
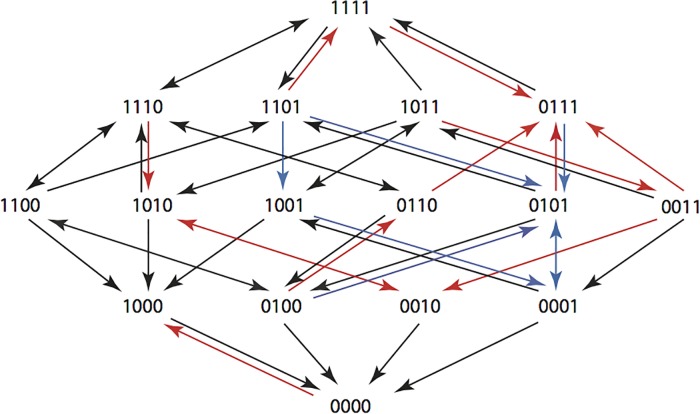
Summary of Optimal 6 Step CPM and EPM Treatment Paths beginning at genotype 1111 and ending at genotype 0000. An arrow indicates that the substitution is included in a path that starts at 1111 and ends at 0000, where the pathway has non-zero probability. Black arrows show substitutions present in six step paths computed using both the CPM and the EPM. Red arrows signify substitutions found only in optimum paths computed using the CPM whereas blue signify substitutions only found using the EPM.

For all sequences of antibiotics of a fixed length (two, three, four, five, and six), we examined the probability that a given genotype is returned to the wild type state. It is worth noting that within these paths, a single genotype can be repeated consecutively with different antibiotics, thus making it possible to have an odd number of steps in the treatment paths when an even number of subtitutions are introduced. For every starting genotype, we found we were able to return to the wild type genotype with a probability between 0.6 and 1.0 when using the CPM model and a probability of 0.375 and 1.0 when using the EPM model. These results are summarized in Tables 6–9 and Fig 17. These results show the number of paths and their probabilities (Tables 6 and 7) and the substitutions selected through the optimal treatment plans (Tables 8–11) for returning to the wild type state from various starting points.

**Table 6:** Maximum Probability and Number of Paths Using CPM.

Starting Genotype	1 Step	No. of paths	2 Step	No. of paths	3 Step	No. of paths	4 Step	No. of paths	5 Step	No. of paths	6 Step	No. of paths
1000	1.0	1	1.0	3	1.0	7	1.0	15	1.0	31	1.0	63
0100	0.617	1	0.617	6	0.617	36	0.617	219	0.617	1360	0.617	8568
0010	0.715	1	0.715	2	0.715	3	0.715	4	0.715	5	0.715	6
0001	0.287	1	0.287	1	0.592	2	0.592	8	0.726	2	0.726	4
1100	-		0.617	3	0.617	18	0.617	108	0.617	657	0.617	4110
1010	-		0.715	1	0.715	6	0.715	27	0.715	112	0.715	453
1001	-		0.559	1	0.559	4	0.726	1	0.726	2	0.729	1
0110	-		0.617	1	0.617	10	0.617	78	0.617	555	0.617	3805
0101	-		0.592	1	0.592	9	0.612	1	0.612	9	0.617	34
0011	-		0.361	1	0.361	9	0.586	2	0.600	2	0.617	8
1110	-		-		0.617	2	0.617	24	0.617	215	0.617	1720
1101	-		-		0.592	2	0.592	24	0.617	12	0.617	252
1011	-		-		0.532	1	0.532	1	0.684	1	0.690	1
0111	-		-		0.586	1	0.600	1	0.617	4	0.617	84
1111	-		-		-	-	0.617	4	0.617	72	0.617	906

**Table 7:** Maximum Probability and Number of Paths Using EPM.

Starting Genotype	1 Step	No. of paths	2 Step	No. of paths	3 Step	No. of paths	4 Step	No. of paths	5 Step	No. of paths	6 Step	No. of paths
1000	1.0	1	1.0	3	1.0	7	1.0	15	1.0	31	1.0	63
0100	0.33	1	0.33	6	0.33	39	0.38	1	0.46	1	0.46	9
0010	0.50	1	0.50	4	0.50	6	0.50	8	0.50	10	0.50	12
0001	0.50	1	0.50	1	0.66	4	0.66	8	0.66	14	0.66	24
1100	-		0.33	27	0.39	1	0.39	1	0.39	4	0.46	5
1010	-		0.50	3	0.50	19	0.58	1	0.58	8	0.59	1
1001	-		0.66	2	0.66	4	0.66	7	0.66	12	0.69	1
0110	-		0.33	1	0.33	10	0.33	81	0.38	1	0.46	1
0101	-		0.29	1	0.38	1	0.46	1	0.46	4	046	1
0011	-		0.25	4	0.25	32	0.50	2	0.50	18	0.50	133
1110	-		-		0.33	2	0.33	24	0.33	221	0.38	6
1101	-		-		0.29	2	0.38	2	0.46	2	0.46	14
1011	-		-		0.33	3	0.33	8	0.39	1	0.52	1
0111	-		-		0.15	1	0.20	8	0.33	4	0.38	6
1111	-		-		-	-	0.33	4	0.38	4	0.46	4

**Table 8:** CPM Additions of Substitutions And Associated β-lactam Antibiotics From Optimal Six Step Treatment Plans (*Maximum Probability for Path).

Substitutions	Drugs associated with substitutions in optimal paths (probability)
0000–1000	CTT(0.38*)
0000–0100	
0000–0010	
0000–0001	
1000–1100	
1000–1010	
1000–1001	
0100–1100	SAM(1.0*)
0100–0110	CTX(1.0*), CPD(1.0*)
0100–0101	
0010–1010	CTT(0.22)
0010–0110	
0010–0011	
0001–1001	AM(1.0*), CTT(0.47), SAM(1.0*)
0001–0101	
0001–0011	
1100–1110	CAZ(0.85*), SAM(0.046), FEP(0.32)
1100–1101	AMP(1.0*),CAZ(0.15), SAM(0.95), FEP(0.68)
1010–1110	CEC(1.0*), CTT(0.47)
1010–1011	
1001–1101	
1001–1011	CTX(0.50*)
0110–1110	FEP(1.0*)
0110–0111	ZOX(1.0*), CXM(0.94), CPD(1.0*)
0101–1101	AMP(1.0*), FEP(1.0*)
0101–0111	CTX(0.58), ZOX(1.0*), CXM(0.59), CPD(0.85)
0011–1011	CTT(0.04)
0011–0111	ZOX(1.0*), CPD(1.0*)
1110–1111	AM(0.90), CRO(0.53), SAM(1.0*), CPD(0.39), FEP(0.72)
1101–1111	AMP(1.0*), SAM(1.0*), FEP(1.0*)
1011–1111	TZP(0.03)
0111–1111	CPD(1.0*)

**Table 9:** CPM Reversions of Substitutions And Associated β-lactam Antibiotics From Optimal Six Step Treatment Plans (*Maximum Probability for Path).

Reversions	Drugs associated with substitutions in optimal paths (probability)
1111–1110	CEC(1.0*), CAZ(0.74), CTT(0.29), CPR(1.0*), TZP(0.15)
1111–1101	AM(1.0*), AMC(1.0*), CAZ(0.26), TZP(0.85)
1111–1011	
1111–0111	ZOX(1.0*), CXM(1.0*)
1110–1100	TZP(0.49*)
1110–1010	AM(0.10), CRO(0.47*), CPD(0.28), FEP(0.28)
1110–0110	CAZ(1.0*), CPR(1.0*), CPD(0.33), TZP(0.51)
1101–1100	
1101–1001	
1101–0101	
1011–1010	TZP(0.30)
1011–1001	TZP(0.92*)
1011–0011	TZP(0.18)
0111–0110	
0111–0101	
0111–0011	
1100–1000	CTT(0.25)
1100–0100	CTX(1.0*), ZOX(1.0*), CXM(1.0*)
1010–1000	CTT(0.53*), TZP(0.49)
1010–0010	ZOX(1.0*), TZP(0.43)
1001–1000	CTX(0.42), CTT(0.56)
1001–0001	
0110–0100	CXM(0.58), TZP(1.0*)
0110–0010	
0101–0100	CTX(0.42), CXM(0.41), CPD(0.15)
0101–0001	
0011–0010	CTT(0.33), TZP(0.45)
0011–0001	CTT(0.20), TZP(0.55)
1000–0000	CPR(1.0*)
0100–0000	AM(0.62*)
0010–0000	TZP(0.71*)
0001–0000	CTT(0.092), CPR(0.14)

**Table 10:** EPM Additions of Substitutions and Associated β-lactam Antibiotics From Optimal Six Step Treatment Plans (*Maximum Probability for Path).

Mutations	β-lactams associated with substitutions in optimal paths (probability)
0000–1000	
0000–0100	
0000–0010	
0000–0001	
1000–1100	
1000–1010	
1000–1001	
0100–1100	SAM(1.0*)
0100–0110	
0100–0101	TZP(1.0*)
0010–1010	
0010–0110	
0010–0011	
0001–1001	AM(1.0*), SAM(1.0*)
0001–0101	TZP(1.0*)
0001–0011	
1100–1110	CTT(1/4)
1100–1101	AMP(1.0*), CPR(1/4)
1010–1110	CTT(1/2)
1010–1011	
1001–1101	
1001–1011	CTX(1/2*)
0110–1110	CTT(1/3)
0110–0111	
0101–1101	AM(1/2), AMC(1/2)
0101–0111	
0011–1011	AMC(1/2*)
0011–0111	
1110–1111	SAM(1.0*)
1101–1111	
1011–1111	CTT(1/3)
0111–1111	SAM(1/2), CPD(1.0*)

**Table 11:** EPM Reversions of Substitutions and Associated β-lactam Antibiotics From Optimal Six Step Treatment Plans (*Maximum Probability for Path).

Reversions	β-lactams associated with substitutions in optimal paths (probability)
1111–1110	CTT(1/3)
1111–1101	AM(1.0*), AMC(1.0*)
1111–1011	
1111–0111	
1110–1100	TZP(1/2*)
1110–1010	
1110–0110	CAZ(1.0*), CPR(1.0*), TZP(1/2)
1101–1100	
1101–1001	CPR(1/3*)
1101–0101	CAZ(1.0*), TZP(1.0*)
1011–1010	CTT(1/3*)
1011–1001	AM(1/2*), CTT(1/3)
1011–0011	
0111–0110	
0111–0101	SAM(1/2*)
0111–0011	
1100–1000	CTT(1/4), CPR(1/4), TZP(1/3*)
1100–0100	CTX(1.0*), ZOX(1.0*), CXM(1.0*)
1010–1000	CTT(1/2*), TZP(1/3)
1010–0010	
1001–1000	CEC(1/2*), CTX(1/2*), CTT(1/2*), CPR(1/2*), TZP(1/3)
1001–0001	CEC(1/2*), CPR(1/2*)
0110–0100	TZP(1.0*)
0110–0010	
0101–0100	CEC(1/2*), AMC(1/2*)
0101–0001	AM(1/2*), CEC(1/2*)
0011–0010	
0011–0001	AMC(1/2*)
1000–0000	CPR(1.0*)
0100–0000	FEP(1/4)
0010–0000	SAM(1/2*), TZP(1/2*)
0001–0000	CEC(1/2*), CPR(1/3), FEP(1/3)

**Fig 18:**
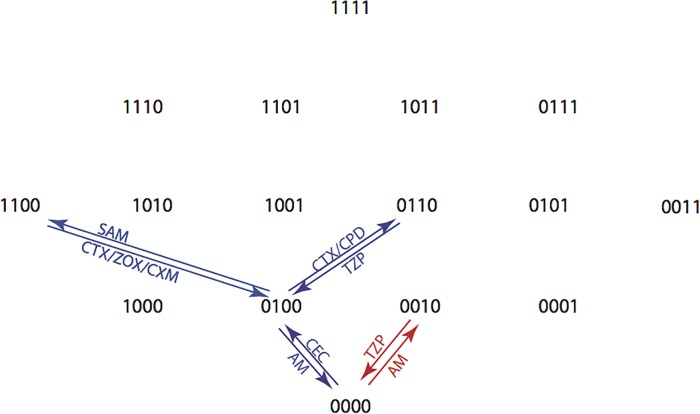
Summary of Optimal CPM 2, 4, and 6 Step Antibiotic Cycles. In this figure, cycles are distinguished from paths in that TEM-1 (0000) is the first and last genotype visited, thus creating circular paths. An arrow indicates a substitution included in a mutational pathway which starts and ends at 0000, where the mutational pathway has a non-zero probability for the optimal treatment cycle. The substitutions that are included in optimal two steps cycles are shown in red. Substitutions that are included in optimal four and six step cycles are shown in blue. Four and six step cycles differ only in the number of substitutions and reversions that occur within each cycle. Their probabilities are identical.

Once returned to the wild type state, we identified cycles that would allow for alternation of antibiotics, and allow for some variation through amino acid substitution, but then rapidly return bacteria to the wild type state (Table 12 and Fig 18). Such cycles were possible for path length of two, four, and six and the probabilities of those paths were respectively 0.704, 0.617, 0.617. We found that in the most probable cases, the genotype varied by only one amino acid substitution before reverting back to the wild type state. However, when treatment plans with lower probabilities are considered, we find that more amino acid substitutions in the genotype are allowed.

**Table 12:** Cyclical Treatment Paths showing Substitutions and Associated β-lactam Antibiotics.

Path length and probability (prob)	0000-0010/ 0010–0000	0000-0100/ 0100–0000	0100-0110/ 0110–0100	0100-1100/ 1100–0100
**2-step (0.70)**				
Cycle 1	AM/TZP			
**4-step (0.62)**				
Cycle 2		CEC/AM	CTX/TZP	
Cycle 3		CEC/AM		SAM/CTX
Cycle 4		CEC/AM		SAM/ZOX
Cycle 5		CEC/AM		SAM/CXM
Cycle 6		CEC/AM	CPD/TZP	
**6-step (0.62)**				
Cycle 7		CEC/AM	CTX/TZP(2x)*	
Cycle 8		CEC/AM	CTX/TZP	SAM/CTX
Cycle 9		CEC/AM	CTX/TZP	SAM/ZOX
Cycle 10		CEC/AM	CTX/TZP	SAM/CXM
Cycle 11		CEC/AM	CTX/TZP, CPD/TZP	

*Steps visited twice along the paths

## Discussion

In this study, we have developed an experimental approach for measuring pleiotropy and a computational mathematical approach for optimizing antibiotic treatment paths. The experimental approach we developed is rapid and high throughput, consistent with previous work [26], and should be applicable to many species of resistant bacteria. The mathematical model we created expresses the problem of antibiotic resistance in general terms, and can therefore be applied to other resistance phenotypes where pleiotropy occurs to identify the antibiotic treatment plans that have the highest probability of reversing the evolution of resistance.

The purpose of this study was to determine whether it is possible to use selective pressures to return TEM-genotypes to the wild type state, as observed in 1963 when TEM-1 was originally isolated. The methods may also be used to select for any particular genotype within our data set. As such, we may select with reasonably high probabilities, resistance genotypes that existed at some prior point in time. To highlight this feature, we have named our software package “Time Machine”.

Once given growth rates of adjacent genotypes, Time Machine returned treatment plans that restored the wild type state as observed in 1963 with probabilities greater than 0.6 when using the CPM model and greater than 3/8 (>0.375) when using EPM. These results suggest that when possible it is desirable to use actual growth rates rather than rough ranking data.

While these treatment methods may have clinical value, we have yet to determine the ideal duration of each therapy. Additionally the antibiotics included in our study may have different applications in the clinic. A further issue is that if new genotypes arise, the treatment plan may fail. The inclusion of more resistance genes in this type of approach may aid in the creation of robust treatment plans that are effective even when unexpected genotypes arise.

The discrete optimization problem motivated by our goal to reverse resistance, or the challenge to build a better time machine, is of independent mathematical interest. Tables 6 and 7 suggest that the maximum probabilities in each row stagnate after a limited number of steps. This is not always the case. We have created an example (see supplemental information) of two substitution matrices on a 3-locus system where the maximum probabilities can be increased indefinitely (S1 Fig).

These results show that great potential exists for remediation of antibiotic resistance through antibiotic treatment plans when pleiotropic fitness costs are known for an appropriate set of antibiotics. While developed using a model of Gram-negative antibacterial resistance, this approach could also be used for Gram-positive bacteria and HIV treatment plans.

## Methods

### Experimental methods

#### Strains and Cultures

We expressed 16 mutant constructs of the bla_TEM_ gene in plasmid pBR322 from strain DH5-αE. The 16 genotypes differ at all combinations of four amino acid residues and have been previously described [14]. We grew them overnight (16 hours) in standing cultures and diluted them to a concentration of 1.9X10^5^ as described elsewhere [14].

We transferred 80 μl of each culture to a 384-well plate with one genotype present in each of the 16 rows. The first 12 wells of each row were antibiotic free (controls) and the last 12 wells contained a single antibiotic at an inhibitory, sublethal concentration. We tested many concentrations and used those that maximized our ability to make comparisons between alleles.

After plating, a membrane is placed over the plate and simultaneously incubated/measured in the Eon Microplate Spectrophotometer at a temperature of 25.1°C for 22 hours. This relatively cool (<37°) temperature is used because degradation of the antibiotics is much slower, while the growth rate of the bacteria is still sufficient to capture the complete exponential period of growth over the duration of the experiment. Overall, we have found that a temperature ~25°C yields more reliable and consistent measurement of growth rates in the presence of antibiotics.

Measurements of cell density (light scattering) at a wavelength of 600 nanometers were automatically collected every 20 minutes after brief agitation to homogenize and oxygenate the culture.

#### Growth Rates

The data obtained from the microplate spectrophotometer is exported to the GrowthRates program to derive the growth rates. In essence, by measuring the optical density at frequent intervals the GrowthRates program can estimate the growth rate,α, through a linear regression algorithm fitting the data from the exponential growth phase. Details can be found in [27] in the section entitled “The Growth Curve” located on pages 233–4. There is not a direct or simple correlation between this method and other methods such as minimum inhibitory (MIC) or disk diffusion testing. The output of this program for the data we collected was a list *f*(*a*_1_),*f*(*a*_2_),…,*f*(*a*_*k*_) of 15 tensors, each of format 2×2×2×2. These are the rows in Table 4. So if *u* ∊ {0,1}^4^ is a genotype, then *f*(*a*_*i*_)_*u*_ is the fitness of genotype *u* in the presence of antibiotic *a*_*i*_. This fitness is a growth rate, so we are here using the letter *f* for a quantity often denoted by α.

One-Way Analysis of Variance (ANOVA) was then used to compare the means of the growth rates obtained, and to determine if there were significant differences between the growth rates of adjacent genotypes.

*Correlated Probability Model (CPM)*: Once the growth rates have been determined under various experimental conditions, the next step is to use them to compute fixation probabilities.

If the (multiplicative) absolute fitnesses *W*_*u*_ and *W*_*v*_ of two neighboring genotypes *u* and *v*, differ by a small quantity then the (additive) relative fitness $\ln\left(\frac{W_{u}}{W_{v}}\right)$ can be approximated by

$$\ln\left(\frac{W_{v}}{W_{u}}\right)=T\left(f_{v}-f_{u}\right)$$ 1

where *T* is the generation time. Using a Taylor series approximation,

$$\ln\left(\frac{W_{v}}{W_{u}}\right)\approx \frac{W_{v}}{W_{u}}-1.$$ 2

If *W*_*v*_ > *W*_*u*_, then

$$p_{u,v}=\frac{f_{v}-f_{u}}{\sum \left(f_{uj}-f_{u}\right)}$$ 3

is the probability for *v* to substitute *u*, where *uj* are the neighbors of *u* with higher fitness than *u* [23].

*Equal Probability Model (EPM)*: According to the EPM model, the probabilities are equal for all beneficial substitutions, so that one needs the fitness graphs only for computing the probabilities. The matrix entry *M*(*f*)_*u*,*v*_ is 1/*N* if genotype *u* has *N* outgoing arrows and there is an arrow from *u* to *v*.

CPM is accurate if fitness differences between genotypes are small, while EPM may provide better estimates if fitness differences are substantial. Indeed, if the fitness effects of all available beneficial mutants exceed some threshold, then fixation probabilities are independent of fitness values [28]. We applied both CPM and EPM, since no complete theory for substitution probabilities exists. Additionally, comparison of two models is useful in learning how sensitive our results are for variation in substitution probabilities.

#### Time Machine Programs

*Optimal antibiotic sequences and pathways of genotypes*: Let *M*(*f*(*a*)) denote the 16×16 transition matrix we derived for the antibiotic labeled *a* (S1 File EPM Prepare and S2 File CPM Prepare). For any sequence *a*_1_,*a*_2_,…*a*_*k*_ of *k* antibiotics, we consider the matrix product *M*(*f*(*a*_1_))*M*(*f*(*a*_2_))*M*(*f*(*a*_3_)). This product is also a 16×16 transition matrix. Its entry in row *u* and column *v* is the fixation probability of genotype *u* mutating to genotype *v* under the antibiotic sequence *a*_1_,*a*_2_,…,*a*_*k*_. That probability is a sum of products of entries in the individual matrices *M*(*f*(*a*_*i*_)), with one sum for each possible pathway of genotypes from *u* to *v*. The Time Machine enumerates all 15^*k*^ antibiotic sequences of length *k*, and it selects all sequences that maximize the entry in row *u* and column *v* of the matrix product (S3 File EPM Run and S4 File CPM Run). In a subsequent step we then analyze these optimal antibiotic sequences, and for each such sequence, we extract the full list of genotype pathways that contribute (S5 File EPM Out and S6 File CPM Out).

We implemented this algorithm in the computer algebra software Maple, and we ran it for *k* = 2,3,4,5,6. The running time of the program is slow because of the exponential growth in the number of sequences. At present we do not know whether an efficient algorithm exists for solving our optimization problem for larger values of *k*.

*Cycles of antibiotics*: We also used this method to compute cyclical treatment paths in which the starting and ending genotypes were the wild type 0000 (S7 File EPM CyclingOut and S8 File CPM CyclingOut). The problem we solved was somewhat different from the previous one, in that we focused on obtaining the maximal probabilities of a cycle that includes some substitutions and then returns to the wild type without halting. Halting means that adjacent genotypes in a mutational pathway coincide, which is undesirable.

## Supporting Information

S1 Fig Locus Model. For any biallelic system and set of drugs, the maximum probabilities for returning to the wild-type depend on how many steps one allows in the treatment plan. The following example demonstrates that the maximum probabilities may increase by the number of steps indefinitely. Consider a three-loci system where the genotypes are ordered as 000; 100; 010; 001; 110; 101; 011; 111: Assume that the starting point is the genotype 100 and that Drugs A and B (see the next page) are available. For the sequence A, the probability for ending at 000 is 0.9, for A-B-A 0.99, for A-B-A-B-A 0.999, and so forth.
(TIFF)

S1 File EPM Prepare. File used to convert growth rate averages into data matrices.
(TXT)

S2 File CPM Prepare. File used to convert growth rate averages into data matrices.
(TXT)

S3 File EPM Run. Input file for computing the probabilities of all possible paths through the landscapes under the EPM Model.
(TXT)

S4 File CPM Run. Input file for computing the probabilities of all possible paths through the landscapes under the CPM Model.
(TXT)

S5 File EPM Out. Number of paths under the EPM model with the greatest probabilities and the antibiotics and genotypes included in those paths.
(TXT)

S6 File CPM Out. Number of circular paths under the CPM model with the greatest probabilities and the antibiotics and genotypes included in those paths.
(TXT)

S7 File EPM Cycling Out. Number of circular paths under the EPM model with the greatest probabilities and the antibiotics and genotypes included in those paths.
(TXT)

S8 File CPM Cycling Out. Number of circular paths under the CPM model with the greatest probabilities and the antibiotics and genotypes included in those paths.
(TXT)
