# Correction: Rational Design of Antibiotic Treatment Plans: A Treatment Strategy for Managing Evolution and Reversing Resistance

**DOI:** 10.1371/journal.pone.0139387

**Published:** 2015-09-24

**Authors:** Portia M. Mira, Kristina Crona, Devin Greene, Juan C. Meza, Bernd Sturmfels, Miriam Barlow

There is an error in the caption for Fig 1, “AMP: Ampicillin 256 μg/ml.” Please see the corrected Fig 1 here.

**Fig 1:**
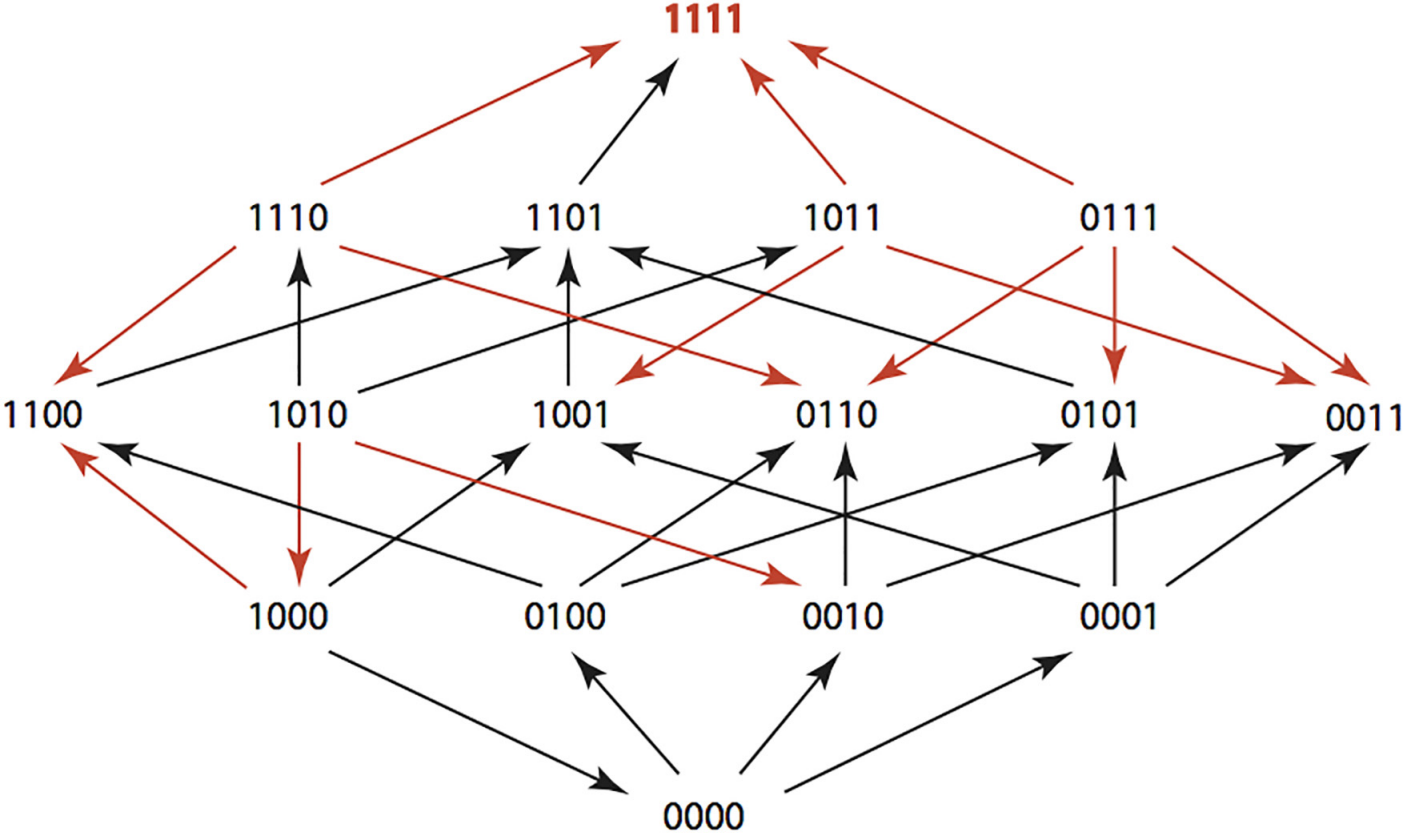
AMP: Ampicillin 2,048 μg/mL

There are errors in the caption for Fig 14, “TZP: Pipercillin / Tazobactam 8.12μg/ml and 8 μg.ml.” Please see the corrected Fig 14 here.

**Fig 14:**
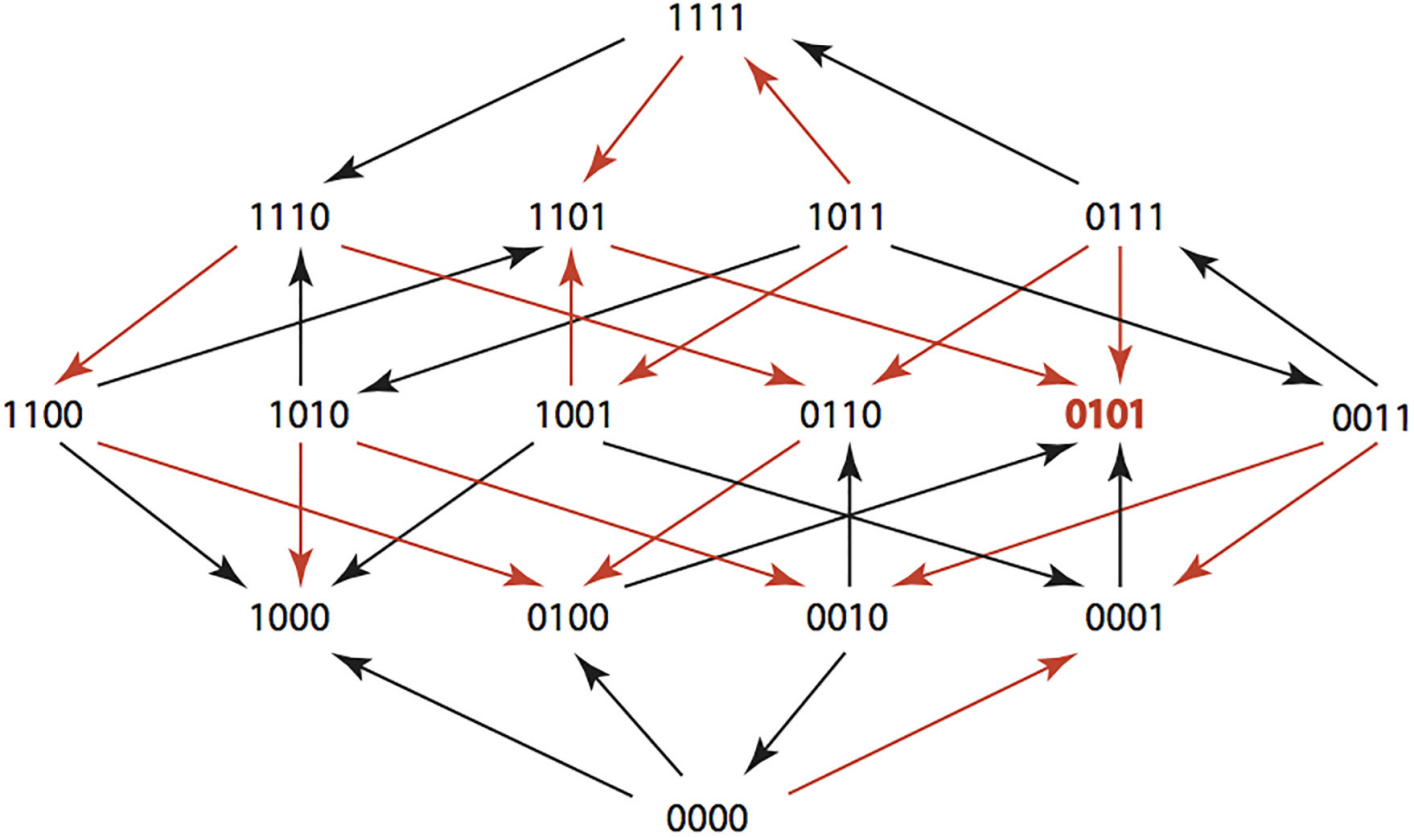
Pipercillin 12 μg/mL Tazobactam 8 μg/mL
