# Supplementary figures and images for: Rational Design of Antibiotic Treatment Plans: A Treatment Strategy for Managing Evolution and Reversing Resistance
Source: PLoS One. 2015 May 6;10(5):e0122283. doi: 10.1371/journal.pone.0122283 (PMC4422678; doi:10.1371/journal.pone.0122283)

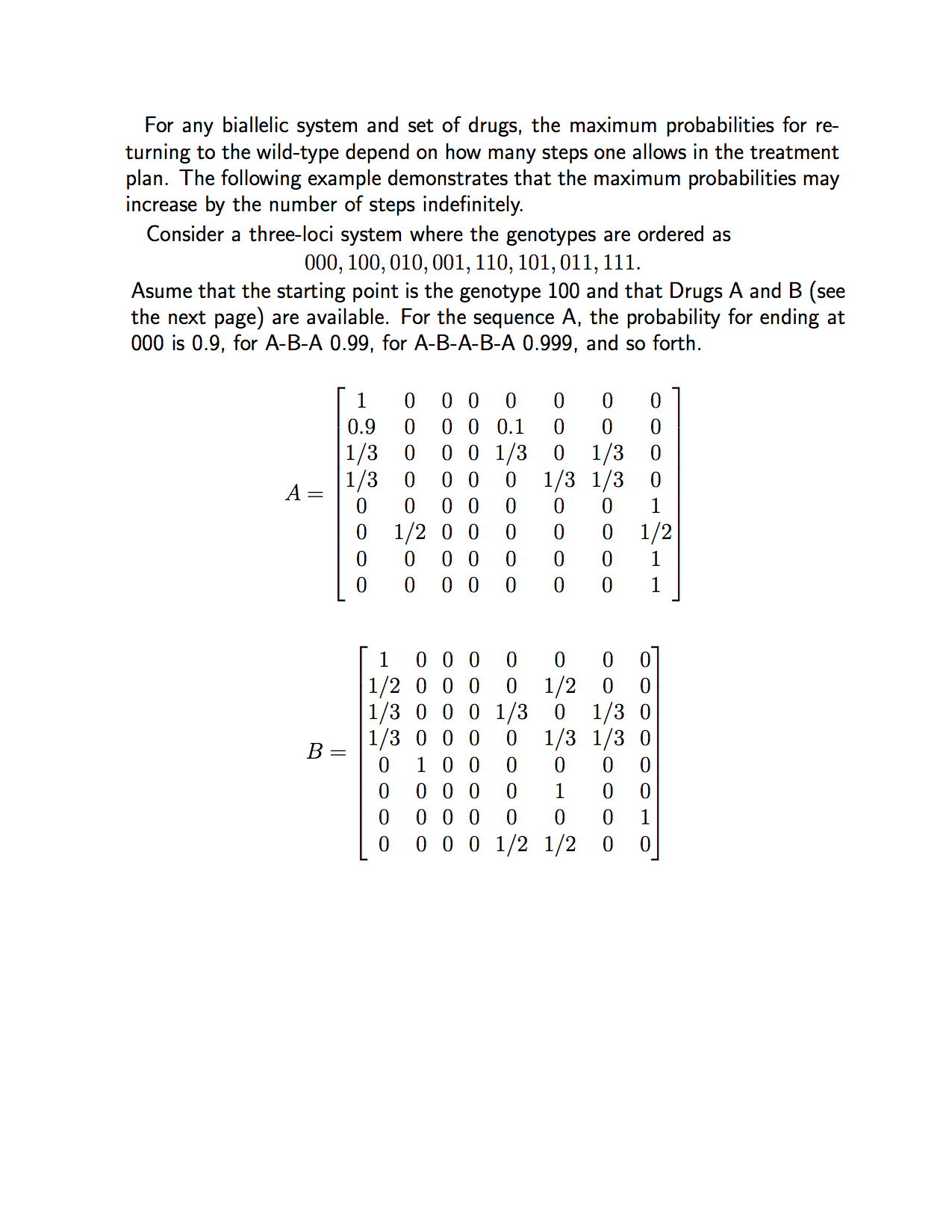

Supplement: S1 Fig — For any biallelic system and set of drugs, the maximum probabilities for returning to the wild-type depend on how many steps one allows in the treatment plan. The following example demonstrates that the maximum probabilities may increase by the number of steps indefinitely. Consider a three-loci system where the genotypes are ordered as 000; 100; 010; 001; 110; 101; 011; 111: Assume that the starting point is the genotype 100 and that Drugs A and B (see the next page) are available. For the sequence A, the probability for ending at 000 is 0.9, for A-B-A 0.99, for A-B-A-B-A 0.999, and so forth. (TIFF) [file pone.0122283.s001.tiff]
